# Efficacy of PARPi re-maintenance therapy for recurrent ovarian cancer

**DOI:** 10.3389/fonc.2024.1512339

**Published:** 2025-01-10

**Authors:** Yulin Wang, Yunjie Yang, Binghong Guo, Xiaoyan Li, Renakezi Tuersun, Ye Cao, Jundong Li, Jihong Liu, Su Li, Tao Liu, Yongwen Huang

**Affiliations:** ^1^ State Key Laboratory of Oncology in South China, Guangdong Provincial Clinical Research Center for Cancer, Sun Yat-sen University Cancer Center, Guangzhou, China; ^2^ Department of Clinical Pharmacology, Guangdong Pharmaceutical University, Guangzhou, China; ^3^ Department of Gynecological Oncology, Cancer Hospital of Shantou University Medical College, Shantou, China; ^4^ Department of Gynecology, The First People’s Hospital of Kashi, Kashi, China

**Keywords:** recurrent ovarian cancer, PARP inhibitors, re-maintenance therapy, prognosis, structural domains of BRCA mutations

## Abstract

**Objective:**

The current clinical data regarding the re-administration of PARPi maintenance therapy in platinum sensitive recurrent ovarian cancer (PSROC) is limited. This study aims to investigate the efficacy and associated factors of PARPi re-maintenance therapy in PSROC patients in China.

**Methods:**

In this study, there were 201 patients with PSROC who had received maintenance therapy previously and achieved complete or partial response after platinum-based chemotherapy upon recurrence. The re-maintenance therapy group (Re-PARPi) and chemotherapy alone group (Chem-A) were categorized based on whether PARPi was reused after recurrence chemotherapy. A propensity-score matching (PSM) analysis was conducted between re-maintenance therapy group (Re-PARPi-P) and chemotherapy alone group(Chem-A-P)to adjust for imbalanced risk factors. The efficacy was evaluated via progression-free survival (PFS) and prognostic factors were also analyzed.

**Results:**

In the PSM subgroup, the median PFS (mPFS) of Re-PARPi-P group (44 cases) and Chem-A-P (44 cases) group were 10.0 months and 6.5 months (HR 1.64, P=0.041) respectively, confirming that re-maintenance therapy was superior to relapse chemotherapy alone. The mPFS was 10.8 months in all patients in the Re-PARPi group (51 cases), with 11.0 months in BRCAm group and 10.2 months in BRCAwt group (P=0.806). Intervals of more than 6 months between two PARPi therapies might improve the efficacy of PARPi re-treatment (mPFS 11.2 months vs. 7.8 months, HR 3.94, P=0.005). Age, BRCA status, number of previous treatment lines, CA125 level prior to re-administration, and other factors were not significantly related to the efficacy of re-maintenance therapy. Patients with a frameshift mutation (p. Ile1824Aspfs3) in the C-terminal domain of BRCA1 germline gene had significantly better efficacy with PARPi re-treatment compared to other groups. Only nonsense mutation (p.Gln1037, p.Cys328, p.Leu1072) occur in BRCA germline gene with re-treatment with PARPi might be suboptimal. The incidence of PARRi re-treatment interruption was 3.9%.

**Conclusion:**

PARPi re-maintenance therapy in PSROC might improve prognosis compared to chemotherapy alone, regardless of their genetic mutation status. Patients with re-maintenance therapy might benefit if the interval between the use of PARP inhibitors exceeded 6 months. The structural domains of BRCA mutations with different sensitivity to PARPi might serve as a promising biomarker for optimizing treatment. Re-treatment with PARPi was well-tolerated.

## Introduction

Ovarian cancer (OC) ranks among the most prevalent gynecological cancers, with approximately 70% of patients diagnosed at advanced stages. Despite receiving standard first-line treatments, approximately 70% of these advanced-stage patients will face recurrence within 1 to 3 years of diagnosis (1). Numerous clinical studies have demonstrated that maintenance therapy with PARP inhibitors (PARPi) significantly reduces the risk of disease progression or mortality in advanced OC patients (2–4). Of these patients, those with BRCA gene mutations are the primary beneficiaries of PARPi maintenance therapy. Additionally, newly diagnosed advanced OC patients exhibiting homologous recombination deficiency (HRD) demonstrate obvious improvements in overall survival (OS) when treated with a combination of olaparib and bevacizumab (5). Since August 2018, various PARPi have received clinical approval in China. Currently, olaparib in combination with bevacizumab is approved for first-line maintenance therapy for newly diagnosed HRD-positive advanced OC, whereas olaparib monotherapy is approved for first diagnosed advanced OC with BRCA-mutation. Niraparib and fuzuloparib are approved for maintenance therapy in newly diagnosed advanced OC, and olaparib, niraparib, and fuzuloparib are approved for platinum-sensitive recurrent epithelial OC (PSROC).

While most patients benefit from first-line PARPi maintenance therapy, some advanced patients still experience tumor progression during or after the completion of PARPi maintenance therapy (6–8). Recent clinical trials have confirmed the feasibility of re-administering PARPi following the initial PARPi therapy. The 2024 edition of the National Comprehensive Cancer Network (NCCN) Guidelines for OC recommends that the PARPi maintenance therapy should be reintroduced only in BRCA-mutated patients with PSROC who have not experienced disease progression during prior PARPi maintenance therapy (Category 2A) (9). The potential for re-administering PARPi maintenance therapy following disease recurrence or progression after initial PARPi therapy has become a focus of attention for Chinese clinicians. This study conducts a retrospective analysis on the efficacy and associated factors of re-administered PARPi maintenance therapy in Chinese patients who experienced disease progression after their initial PARPi maintenance therapy, achieved complete response (CR) or partial response (PR) after chemotherapy, and subsequently received PARPi maintenance therapy again. The study aims to evaluate the feasibility of reintroducing PARPi maintenance therapy.

## Materials and methods

### Study population

This study included patients with recurrent OC who were treated at the Gynecologic Oncology Department of Sun Yat-sen University Cancer Center between 2018-08-01 and 2023-12-31. These patients had previously received maintenance therapy with olaparib or niraparib and were retreated with platinum-based chemotherapy after tumor progression. The study was approved by the Ethics Committee of Sun Yat-sen University Cancer Center (Ethics Approval Number: SL-B2023-100-01), a waiver for informed consent was granted, and all enrolled patients were not required to sign informed consent forms.

### Inclusion criteria

(1) Age ≥ 18 years; (2) Histopathologically confirmed primary ovarian epithelial cancer, peritoneal cancer, or fallopian tube cancer; (3) Performance status (PS) score of 0 or 1; (4) Prior monotherapy maintenance with olaparib or niraparib; (5) A complete or partial response to the most recent chemotherapy; (6) Availability of complete clinical and pathological data.

### Exclusion criteria

(1) Concurrent diagnosis of other primary malignancies within the past 5 years; (2) Irregular use of PARPi (defined as receiving PARPi therapy for less than 28 days or discontinuing therapy for over 28 days at a time); (3) Concurrent use of other anti-tumor drugs during previous maintenance therapy; (4) Presence of severe comorbidities.

### Data collection

Clinical data were collected for all eligible participants including diagnosis age, PS score, family cancer history, occurrence of secondary cytoreductive surgery, surgical outcomes from secondary cytoreductive surgery, tumor histological type, tumor differentiation grade, disease stage (based on the 2014 International Federation of Gynecology and Obstetrics [FIGO] surgical-pathological staging criteria), genetic test results, anti-tumor treatment regimens, maintenance therapy regimens, tumor progression details, and treatment-emergent adverse events (TEAEs).

### Follow-up

Follow-up was conducted via outpatient visits, telephone calls, and emails until 2024-05-30. Data on patient survival status, time to disease progression, date of death, and cause of death were recorded.

### Statistical analysis

All enrolled patients were categorized into two groups: the PARPi re-maintenance therapy group(Re-PARPi)and the chemotherapy alone group(Chem-A), based on whether they reused PARPi maintenance therapy after relapse chemotherapy. The primary endpoint of this study was progression-free survival (PFS), defined as the interval from the completion of re-chemotherapy to the time of tumor progression or the latest follow-up. Treatment responses were evaluated according to the Response Evaluation Criteria in Solid Tumors (RECIST) 1.1, with tumor burden changes serving as the reference. Short-term treatment outcomes were classified as CR, PR, stable disease (SD), or progressive disease (PD) (10).

All data collected were entered into an Excel database and analyzed using SPSS version 26.0. A descriptive statistical analysis focused on clinical pathological characteristics and treatment efficacy. Continuous variables were reported using median ± interquartile range (IQR), while categorical variables were reported using rates and proportions (%).

Propensity score matching (PSM) analysis was performed to balance the variables affecting the treatment selection among different treatment options. PSM procedure was performed using SPSS software with the option of extract matched control. A caliper width of 0.05 was applied to ensure optimal matching precision.

Kaplan-Meier survival analysis was conducted to estimate the survival curves. Univariate and multivariate Cox regression models were utilized to identify factors influencing the prognosis of PARPi maintenance therapy. Wilson’s continuity correction was used to calculate the 95% confidence interval (95% CI). All analyses operated at a significance level of 5%.

## Results

### Baseline characteristics of patients

**Figure 1:**
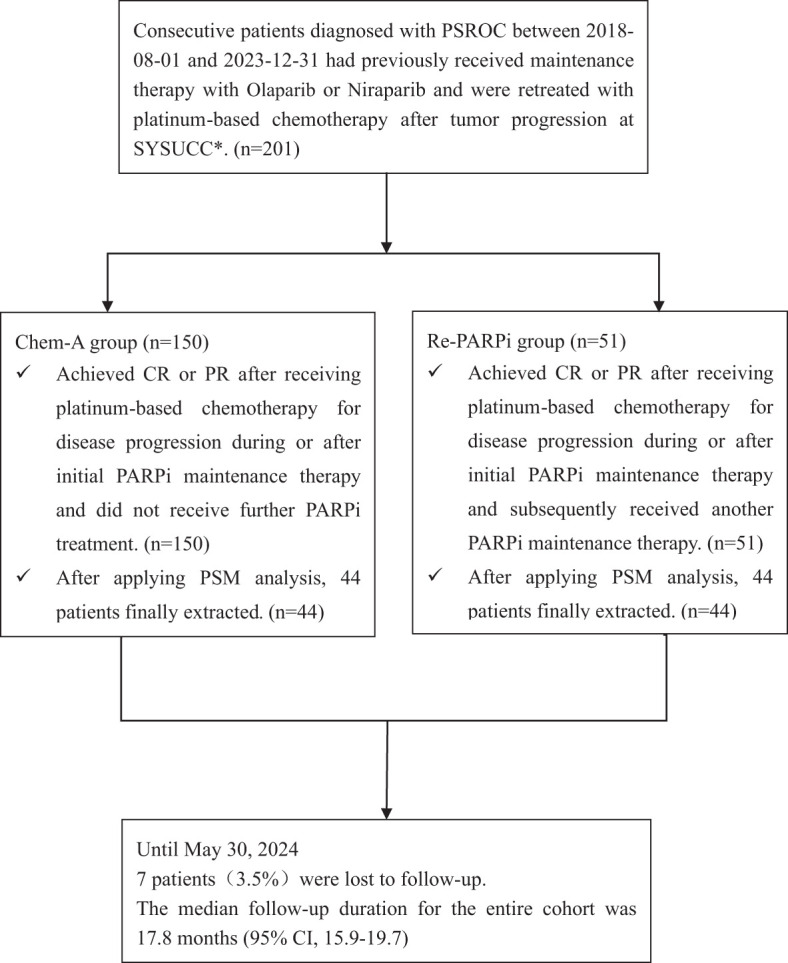
Procedures for patients selection and follow-up. PSROC, platinum sensitive recurrent ovarian cancer; SYSUCC, Sun Yat-sen University Cancer Center; CR, complete response; PR, partial response; PSM, Propensity score matching.

In this retrospective analysis, 201 patients were included. There were 150 contemporaneous patients in the Chem-A group who received platinum-based chemotherapy for disease progression during or after initial PARPi maintenance therapy. They achieved CR or PR and did not receive further PARPi treatment. In comparison, the Re-PARPi group consisted of 51 patients subsequently received another PARPi maintenance therapy after platinum-based chemotherapy (**Figure 1**).

Until 2024-05-30, the median follow-up duration for the entire cohort was 17.8 months (95% CI, 15.9-19.7), with 7 patients(3.5%)were lost to follow-up. The median age of the patients was 54.34 ± 8.92 years.

In the Re-PARPi group, the median follow-up duration was 22.7 months(95% CI, 18.1-28.0). Of 51 patients, 11 (21.6%) had passed away, and 14 (27.5%) were still receiving maintenance therapy. The median duration of initial PARPi maintenance therapy was 12.2 months (95% CI, 9.5-14.5). During initial PARPi maintenance therapy, 43 patients (84.3%) experienced disease recurrence, with a median recurrence time of 10.0 months (95% CI, 8.2-11.8), while 8 patients (15.7%) relapsed after completing initial PARPi maintenance therapy, with a median relapse time of 29.1 months (95% CI, 19.3-39.7). The median interval between the two PARPi treatments was 7.4 months (95% CI, 6.0-8.0). Prior to restarting PARPi maintenance therapy, 10 patients (19.6%) developed visceral metastases, of which 5 were liver metastases, 3 were lung metastases, and 2 were multi-organ metastases involving the liver, lung, and spleen.

The median follow-up duration in the Chem-A group was 16.4 months(95% CI,13.5-19.2), with 50 patients (33.3%) experiencing mortality. The median duration of initial PARPi maintenance therapy was 8.0 months (95% CI, 6.2-9.8).

The baseline characteristics between Re-PARPi group and Chem-A group were uneven distributed, the median follow-up duration were 22.7 months and 16.4 months respectively (P=0.009). Furthermore, the proportion of patients with initial PARPi treatment time <6 months varied from 13.7% in the Re-PARPi group to 34.7% in the Chem-A group (P=0.005) (**Table 1**). After applying PSM analysis, 88 patients were finally extracted [44 pairs of well-matched case-control, i.e., the re-maintenance therapy group (Re-PARPi-P) and chemotherapy alone group(Chem-A-P)]. The PSM analysis balanced the risk factors between these two groups (**Table 1**).

**Table 1:** Baseline characteristics in overall population and in propensity score matched population.

Characteristics	Overall population			Propensity scores matched population		
	Maintenance Therapy (n=51) (%)	Chemotherapy (n=150) (%)	P-value	Maintenance Therapy (n=44) (%)	Chemotherapy (n=44) (%)	P-value
Median follow-up in months (95%CI)	22.7 (18.1-28.0)	16.4 (13.5-19.2)	0.009	21.3 (14.7-27.8)	20.0 (9.3-30.6)	0.521
Age, median (years)	53.57 ± 8.36	54.61 ± 9.11	0.055	54.39 ± 8.30	52.27 ± 8.97	1.000
<65	48 (94.1)	125 (83.3)		41 (93.2)	41 (93.2)	
≥65	3 (5.9)	25 (16.7)		3 (6.8)	3 (6.8)	
Family history of tumors			0.592			0.803
No	39 (76.5)	120 (80.0)		34 (77.3)	33 (75.0)	
Yes	12 (23.5)	30 (20.0)		10 (22.7)	11 (25.0)	
BRCA status			0.239			0.622
BRCAm	15 (29.4)	32 (21.3)		12 (27.3)	10 (22.7)	
BRCAwt	36 (60.8)	118 (78.7)		32 (72.7)	34 (77.3)	
Pathological type						1.000
HGSC	48 (94.1)	138 (92.0)	0.619	43 (97.7)	43 (97.7)	
Others	3 (5.9)	12 (8.0)		1 (2.3)	1 (2.3)	
Previous treatment lines			0.826			0.140
=2	33 (64.7)	89 (59.3)		36 (81.8)	30 (68.2)	
≥3	18 (35.3)	61 (40.7)		8 (18.2)	14 (31.8)	
Initial PARPi treatment time			0.005			0.764
<6 months	7 (13.7)	52 (34.7)		7 (15.9)	6 (13.6)	
≥6 months	44 (86.3)	98 (65.3)		37 (84.1)	38 (86.4)	
Relapse time of initial PARPi treatment			0.340			0.764
During	43 (84.3)	134 (89.3)		37 (86.3)	38 (86.4)	
After	8 (15.7)	16 (10.7)		7 (13.7)	6 (13.6)	
Secondary surgery			0.826			0.877
R0	14 (27.5)	32 (21.3)		11 (25.0)	9 (20.5)	
R1/R2	2 (3.9)	9 (6.0)		2 (4.5)	2 (4.5)	
Not secondary surgery	35 (68.6)	109 (72.7)		31 (70.5)	33 (75.0)	
Chemotherapy region			0.333			0.667
Chemotherapy	23 (45.1)	91 (60.7)		24 (54.5)	26 (59.1)	
Chemotherapy+Bevacizumab/Erlotinib	28 (54.9)	59 (39.3)		20 (40.9)	18 (40.9)	
Chemotherapy remission			0.338			0.453
CR	13 (25.5)	49 (32.7)		12 (27.3)	9 (20.5)	
PR	38 (74.5)	101 (67.3)		32 (72.7)	35 (79.5)	
**Visceral metastasis**			0.952			0.777
No	41 (80.4)	120 (80.0)		36 (81.8)	37 (84.1)	
Yes	10 (19.6)	30 (20.0)		8 (18.2)	7 (15.9)	
CA125 after Chemotherapy			0.058			0.467
<35 U/ml	35 (68.4)	122 (81.3)		31 (70.5)	34 (77.3)	
≥35 U/ml	16 (31.4)	28 (18.7)		13 (29.5)	10 (22.7)	

CI, confidence interval; HGSC, high-grade serous carcinoma; CR, complete response; PR, Partial response.

### Progression-free survival of recurrent ovarian cancer

**Figure 2:**
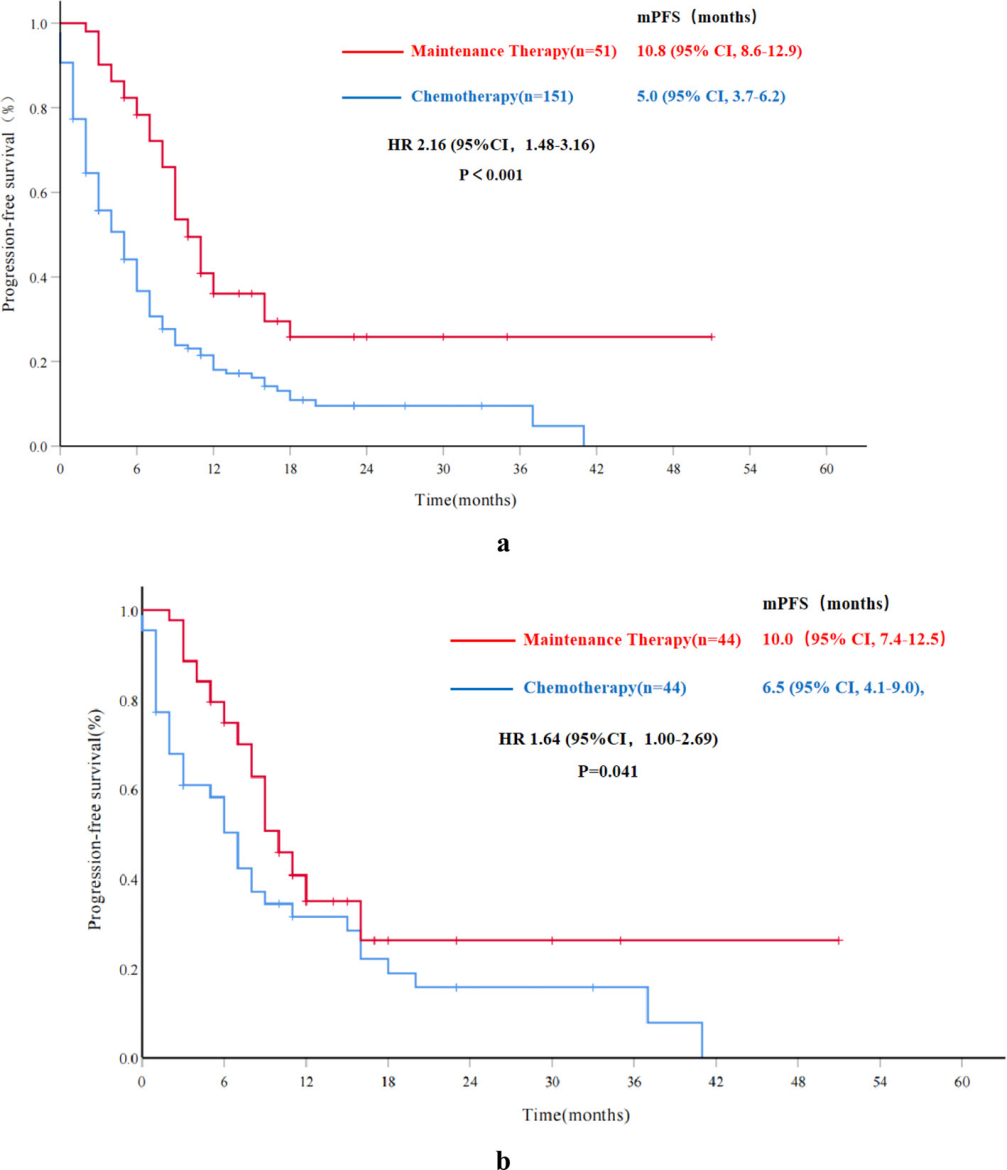
Progression-free survival in overall and propensity score matched cohorts. **(A)** Progression-free survival for the overall cohort; **(B)** Progression-free survival for the propensity score matched cohorts. mPFS, median progression-free survival; HR, hazard ratio; CI, confidence interval.

By the last follow-up, 160 patients (79.6%) of the entire sample population had experienced tumor progression. The Chem-A group had a higher relapse rate with 126 patients (126/150, 84.0%) recurring compared to 34 patients (34/51, 66.7%) in the Re-PARPi group. The mPFS for the Chem-A group and Re-PARPi group was 5.0 months (95% CI, 3.7-6.2) and 10.8 months (95% CI, 8.6-12.9) respectively, with significant statistical difference (HR 2.16, 95%CI 1.48-3.16, P<0.001) (**Figure 2A**).

In the PSM subgroup, mPFS for the Chem-A-P group was 6.5 months (95% CI, 4.1-9.0), while the Re-PARPi-P group was 10.0 months(95% CI, 7.4-12.5). The PFS benefit was statistically significant in the Re-PARPi-P group compared with the Chem-A-P group (HR 1.64, 95%CI 1.00-2.69, P=0.041) (**Figure 2B**).

### Factors associated with the efficacy of PARPi Re-maintenance therapy

In multivariate analysis, the median PFS of Re-PARPi group was superior to Chem-A group (HR 1.99, 95%CI 1.42-2.78, p < 0.001). Besides, patients who achieved CR after the most recent chemotherapy had better outcomes compared with PR patients (HR 1.92, 95% CI 1.34-2.74, P<0.001) (**Table 2**).

**Table 2:** Multivariate analysis for PFS in overall and PSM cohort.

Characteristics	Risk Group	Overall population				Propensity scores matched population			
		Univariate		Multivariate		Univariate		Multivariate	
		P-value	HR (95%CI)	P-value	HR (95%CI)	P-value	HR (95%CI)	P-value	HR (95%CI)
Re-PARPi	No (vs Yes)	<0.001	1.99 (1.42-2.78)	<0.001	2.36 (1.60-3.49)	0.018	1.55 (0.94-2.56)	0.035	1.73 (1.04-2.87)
Chemotherapy remission	PR (vs CR)	0.003	1.53 (1.10-2.13)	<0.001	1.92 (1.34-2.74)	0.036	1.79 (1.06-3.03)	0.103	1.79 (0.90-3.56)
Initial PARPi treatment time	<6 (vs≥6) months	0.041	1.20 (0.83-1.73)	0.450	1.15 (0.80-1.64)	0.512	0.94 (0.48-1.82)		
Relapse time of initial PARPi treatment	During (vs after)	0.071	1.76 (1.09-2.82)	0.121	1.56 (0.89-2.72)	0.245	1.75 (0.92-3.34)		
Age (years)	≥65(vs<65)	0.384	1.34 (0.63-2.87)			0.648	1.27 (0.46-3.50		
Family history of tumors	Yes (vs No)	0.163	1.26 (0.83-1.91)			0.087	1.44 (0.76-2.75)	0.101	1.64 (0.91-2.94
BRCA status	BRCAwt (vs BRCAm)	0.998	1.02 (0.69-1.49)			0.447	0.95 (0.53-1.72)		
Pathological type	HGSC (vs Others)	0.170	0.65 (0.32-1.32)			0.942	0.68 (0.13-3.69)		
Previous treatment lines	≥3 (vs 2)	0.526	1.06 (0.76-1.48)			0.251	1.25 (0.73-2.15)		
Secondary surgery	No (vs Yes)	0.341	1.19 (0.84-1.68)			0.078	1.46 (0.88-2.45)	0.817	1.04 (0.75-1.45)
Chemotherapy region	Chemotherapy(vs+Bevacizumab/Erlotinib)	0.991	1.05 (0.76-1.46)			0.296	1.32 (0.80-2.19)		
CA125 after Chemotherapy	≥35 (vs<35) U/ml	0.188	1.15 (0.79-1.67)			0.597	1.38 (0.78-2.43)		
Visceral metastasis	Yes (vs No)	0.333	1.01 (0.69-1.49)			0.878	1.34 (0.73-2.49)		

PSM, Propensity score matching; PFS, progression-free survival; HR, hazard ratio; CI, confidence interval; HGSC, high-grade serous carcinoma; CR, complete response; PR, Partial response.

In the PSM subgroup, mPFS was better in the Re-PARPi-P group than Chem-A-P group (HR 1.73, 95% CI 1.04-2.87, P=0.035). Notably, whether PARPi was re-maintenance was the only independent factor of PFS (**Table 2**).

**Figure 3:**
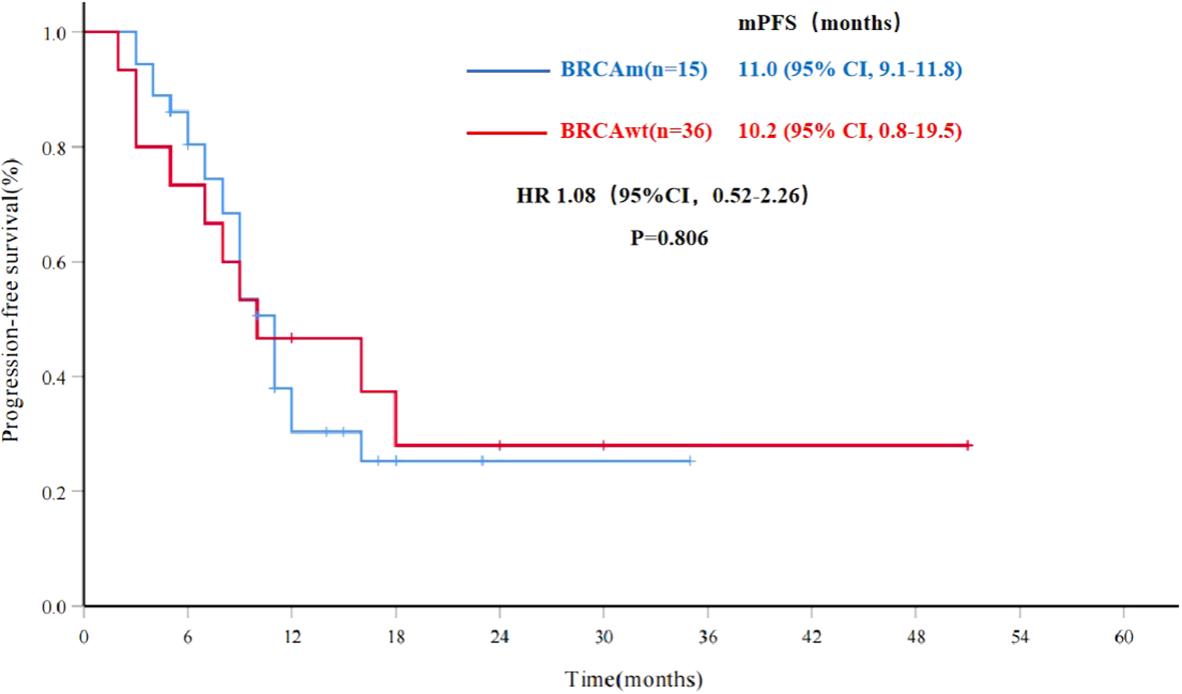
Progression-free survival for PARPi re-maintenance therapy by BRCA status (n=51). mPFS, median progression-free survival; BRCAwt, BRCA wild type; BRCAm, BRCA mutations; HR, hazard ratio; CI, confidence interval.

To explore potential factors influencing the efficacy of PARPi re-maintenance therapy, further analysis was conducted. The mPFS was 10.8 months (95% CI, 8.6-12.9) for all patients in the Re-PARPi group (51 cases), and 11.0 months (95% CI, 9.1-11.8) and 10.2 months (95% CI, 0.8-19.5) for the BRCAm and BRCAwt populations respectively. There was no significant statistical difference between the results in these two groups (HR 1.08, 95%CI, 0.52-2.26, P=0.806) (**Figure 3**).

**Figure 4:**
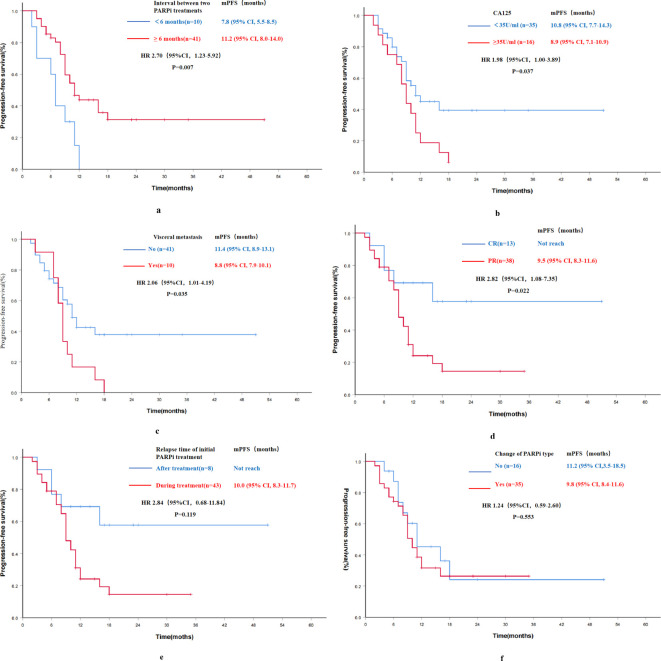
Progression-free survival results of univariate analysis for PARPi re-maintenance therapy group (n=51). **(A)** Progression-free survival by the interval between two PARPi treatments; **(B)** Progression-free survival by CA-125 levels before re-maintenance therapy; **(C)** Progression-free survival by the status of visceral metastasis; **(D)** Progression-free survival by the best response to the most recent chemotherapy before re-maintenance therapy; **(E)** Progression-free survival by the relapse time of initial PARPi treatment; **(F)** Progression-free survival by the change of PARPi type; mPFS, median progression-free survival; HR, hazard ratio; CI, confidence interval.

In the Re-PARPi group, a univariate analysis was performed to identify factors that might affect the efficacy of PARPi re-maintenance therapy in PSROC patients. The results exhibited that the interval between two PARPi treatments, CA-125 levels before re-maintenance therapy, the presence or absence of visceral metastasis, and the best response (CR or PR) to the most recent chemotherapy before re-maintenance therapy were associated with the efficacy of PARPi re-maintenance (**Figure 4**). However, factors such as age, family history of malignancy, BRCA mutation status, number of previous chemotherapy lines, duration of previous PARPi exposure, and type of previously used PARPi were not significantly associated with the efficacy of re-treatment (**Table 3**).

**Table 3:** Progression-free survival results of multivariate analysis for PARPi re-maintenance therapy group (n=51).

Characteristics	Risk Group	PARPi re-maintenance therapy group (n=51)			
		Univariate		Multivariate	
		P-value	HR (95%CI)	P-value	HR (95%CI)
Time between PARPi treatment (months)	<6 (vs≥6)	0.007	2.70 (1.23-5.92)	0.005	3.94 (1.52-10.3)
Chemotherapy remission	PR (vs CR)	0.022	2.82 (1.08-7.35)	0.300	1.76 (0.60-5.13)
Visceral metastasis	Yes (vs No)	0.035	2.06 (1.01-4.19)	0.812	1.10 (0.49-2.48)
CA125 after Chemotherapy	≥35 U/ml (vs<35 U/ml)	0.037	1.98 (1.00-3.89)	0.082	2.33 (0.90-6.05)
Age (years)	≥65 (vs<65)	0.181	2.15 (0.65-7.09)		
Family history of tumors	Yes (vs No)	0.789	1.11 (0.50-2.45)		
BRCA status	BRCAwt (vs BRCAm)	0.847	1.07 (0.51-2.26)		
Pathological type	HGSC (vs Others)	0.443	0.64 (0.19-2.12)		
Initial PARPi treatment time	<6 (vs≥6)	0.538	1.37 (0.48-3.89)		
Relapse time of initial PARPi treatment	During (vs after)	0.119	2.84 (0.68-11.8)		
Previous treatment lines	≥3 (vs 2)	0.267	1.52 (0.70-3.28)		
Secondary surgery	No (vs Yes)	0.341	1.11 (0.75-1.63)		
Chemotherapy region	Chemotherapy (vs + Bevacizumab/Erlotinib)	0.223	1.49 (0.76-2.93)		

HR, hazard ratio; CI, confidence interval; HGSC, high-grade serous carcinoma; CR, complete response; PR, Partial response

Further multivariate analysis for the Re-PARPi group indicated a significantly improvement in mPFS of 11.2 months in patients with an interval of ≥6 months between the two PARPi treatments compared with 7.8 months in those with an interval of <6 months (HR 3.94, 95% CI 3.94-10.3, P=0.005). The independent factor affecting PFS in PARPi re-maintenance therapy was the interval between two PARPi treatments (**Table 3**).

### Molecular analysis

Analysis of mutation status revealed that PSROC patients with a frameshift mutation (p. Ile1824Aspfs3) in the C-terminal BRCT domain of the BRCA1 germline gene had significantly higher efficacy of PARPi re-treatment compared with the other groups. If only nonsense mutation (p. Gln1037, p. Cys328, p. Leu1072) occur in the BRCA germline gene, the efficacy of re-treatment with PARPi in these patients might be suboptimal. Furthermore, compared to the efficacy of previous PARP inhibitor treatment, it was hypothesized that this could be a potential factor contributing to PARP inhibitors resistance (**Table 4**).

**Table 4:** Major genetic profiling detected in tumor tissues for PARPi re-maintenance therapy group.

Patient	PFS1 (months)	PFS (months)	Mutant gene	Mutation characteristics
1	3.7	50.6	BRCA1	p. Glu1115*fs*1
2	7.3	30.2	BRCA1	p. Ile1824Aspfs*3
3	10.0	18.1	RAD51D	p. Lys91Ilefs*13
4	30.6	16.7	MLH3	p. Ile709Val
5	22.5	16.5	BRCA1	p.I1159*fs*1
6	17.9	10.8	BRIP1	p.K1670*
7	12.5	10.8	PMS1	p. Thr451Met
8	8.1	10.8	MSH6	p. His501Tyr
9	18.1	9.3	TP53	p. Tyr163Cys
10	23.8	9.3	TP53	p. Val173Leu
11	13.0	8.9	BRCA1	p. Leu502Alafs*2
12	14.1	8.9	PIK3CA	p. His1047Arg 29.4%
13	12.6	8.8	BRCA1	BRCA1 p. Arg1751* 58.1% KRAS p. Gly12Val 1.7%
14	8.3	8.5	FANCD2	p.1279-2Ala>Thr
15	14.5	7.1	BRCA1	p. Glu1038Leufs*5
16	19.6	3.3	BRCA2	p. Gln1037*
17	17.0	2.9	BRCA1	p. Cys328*
18	21.0	2.8	BRCA1	p. Leu1072*

Green indicates missense. Purple indicates frameshift. Yellow indicates splice. Orange indicates somatic cell mutation.

*p., amino acid variation at the protein level; “fs”, frame shift; * denotes nonsense.

PFS, progression-free survival, the interval from the completion of re-chemotherapy to the time of tumor progression or the latest follow-up.

PFS1, the interval from the start of initial PARPi maintenance therapy to the time of tumor first progression.

### Treatment-emergent adverse events

In the Re-PARPi cohort, the most common TEAEs in patients receiving re-maintenance therapy was anemia (37.3%). The majority of TEAEs were grades 1-2. No TEAEs results in death and there was no treatment termination due to TEAEs (**Table 5**).

**Table 5:** Summary of TEAEs for PARPi re-maintenance therapy group (n=51).

TEAES	Any grade, n (%)	Grade≥3, n (%)
Any	35 (68.6)	7 (13.7)
Anemia	19 (37.3)	5 (9.8)
Leukopenia	14 (27.5)	2 (3.9)
Neutropenia	11 (21.6)	3 (5.9)
Thrombocytopenia	7 (13.7)	2 (3.9)
Vomiting	7 (13.7)	0
Abdominal pain	4 (7.8)	0
Urinary tract infection	2 (3.9)	0
Leading to dose modification	5 (9.8)	
Leading to treatment interruption	2 (3.9)	

TEAEs, Treatment-emergent adverse events.

During PARPi re-maintenance treatment period, 5 patients (9.8%) had their PARPi dose adjusted because of grade 3 or higher TEAEs, 2 patients (3.9%) experienced PARPi re-treatment interruptions, 1 for 12-days discontinuations due to grade 4 thrombocytopenia and 1 for 14-days interruption due to grade 3 anemia (**Table 5**).

## Discussion

In recent years, multiple phase III clinical studies have demonstrated that PARPi can effectively improve PFS and OS in patients with advanced OC (5, 11–14). The introduction of PARPi has significantly transformed the treatment landscape for OC. However, the increase in clinical use of PARPi determines the optimal treatment strategy after progression on PARPi therapy has become a clinical challenge. The OReO/ENGOT Ov-38 study, the first randomized, double-blind, placebo-controlled phase IIIB study exploring re-treatment with PARPi, focused on PFS as the primary endpoint. The study demonstrated a significant improvement in PFS in PSROC patients who had previously received PARPi maintenance therapy and were re-treated with olaparib compared to those who received placebo, with mPFS of 4.3 months vs. 2.8 months in the BRCAm group and 5.3 months vs. 2.8 months in the BRCAwt group (15).

A retrospective study of 26 patients with recurrent epithelial ovarian cancer who received PARPi re-maintenance therapy displayed an mPFS of 7.4 months for BRCAm patients and 4.5 months for BRCAwt patients. No statistically significant difference was observed between the two groups (16). A real-world study in China involving 49 patients analyzed the time to next treatment (TTNT) benefit of PARPi re-treatment, showed that the TTNT1 (16.4 months vs. 12.1 months, P=0.052) and TTNT2 (7.3 months vs. 5.0 months, P=0.555) were longer in BRCAm patients compared to BRCAwt patients. However, the difference was not statistically significant (17). These real-world studies suggest that PARPi re-treatment provides some PFS benefit in patients with recurrent ovarian cancer, but the impact of prognostic factors on PFS benefit remains unclear.

The first PARPi, olaparib, was approved in China in August 2018 for the maintenance treatment of PSROC. Both olaparib and niraparib have been used for a relatively short period among Chinese patients, and the feasibility of PARP inhibitors for re-maintenance therapy is currently under exploration. Before a consensus is reached on the conditions of PARPi re-treatment, Sun Yat-sen University Cancer Center re-administers PARPi maintenance therapy to certain OC patients who had achieved CR or PR after chemotherapy for recurrent disease according to Chinese guidelines and clinical treatment needs.

In the PSM analysis of PSROC patients who experienced tumor progression after initial PARPi treatment, re-maintenance therapy was associated with a significantly longer PFS compared to relapse chemotherapy alone (mPFS 10.0 months vs. 6.5 months, HR 1.64, P=0.041). The results also presented that mPFS for all patients receiving re-maintenance therapy as well as the BRCAm and BRCAwt cohorts, were 10.8 months, 11.0 months, and 10.2 months respectively, which is consistent with the benefits observed in previous retrospective studies. Regardless of BRCA mutation status, the PSROC patients in this study benefited from PARPi re-maintenance therapy, with no significant difference in benefit between BRCAm and BRCAwt patients.

The platinum-free interval (PFI) is widely used to predict the response to subsequent chemotherapy and its survival outcomes in ovarian cancer patients. However, data regarding the effect of the PARPi-free interval on the sensitivity of OC patients to receive PARPi re-treatment are limited. This study suggested that patients with PARPi-free intervals of ≥6 months had significantly greater benefit from PARPi re-treatment compared to those with a PARPi-free interval of <6 months (HR 3.94, P=0.005), which indicated that patients with PSROC who had paused PARPi therapy for more than 6 months could be considered for PARPi re-treatment if there were no contraindications. CA-125 is widely used for prognosis and efficacy evaluation in epithelial OC. A retrospective study involving 10,594 patients with epithelial OC found that higher pre-treatment CA-125 levels were associated with lower patient survival (18). In the OReO study, CA-125 levels (HR 1.50, P=0.015) and the presence of visceral metastases at baseline (HR 2.04, p<0.0001) were the best predictors of patient prognosis (19). Consistent with previous studies, univariate analysis found that patients with pre-treatment CA-125 levels ≥35 U/ml (mPFS 8.9 months) had less PFS benefits compared to patients with CA-125 levels <35 U/ml (mPFS 10.8 months) in our study, but multivariate analysis showed no significant difference, which may be due to limited sample size. However, this observation suggests that pre-treatment CA-125 levels may influence the efficacy of PARPi maintenance therapy and implies that PARPi re-maintenance therapy may be more suitable for PSROC patients with normal pre-treatment CA-125 levels.

The 2024 NCCN guidelines for OC recommend that the reintroduction of maintenance therapy with PARPi is conditional on the absence of disease progression during initial PARPi maintenance therapy. In the re-maintenance therapy group of our study, 8 patients (15.7%) who did not experience disease progression during their initial PARPi maintenance therapy did not achieve mPFS with re-treatment. Although their mPFS was longer compared to patients who experienced disease progression during the initial PARPi maintenance therapy (mPFS 10.0 months), the difference was not statistically significant (HR 2.84, P=0.119), which may be due to the small sample size. This suggests that patients who did not experience disease progression during initial PARPi maintenance therapy may benefit more from re-treatment. In this study, 16 patients (31.4%) who did not change the type of PARPi during re-maintenance therapy had an mPFS of 11.2 months, compared to 9.8 months for those who changed PARPi type. There was no significant statistical difference emerged between the two groups. The mechanisms underlying PARPi resistance include overexpression of the drug pump P-glycoprotein, genetic reversion of BRCA1 or BRCA2 mutations, reactivation of DNA damage repair, and activity of BRCA1 or BRCA2 sub-alleles. The complexity of PARPi resistance mechanisms suggests that changing the type of PARPi during re-maintenance therapy may not significantly affect efficacy.

A retrospective analysis involving 79 cases with BRCA mutations revealed that the most common structural domains of BRCA1 mutations in high-grade serous ovarian cancer (HGSC) were the BRCT (C-terminal) domain (15 cases, 31%) and the DNA Binding Domain (DBD) (13 cases, 27%). Mutations in these BRCA DNA binding domain were associated with high sensitivity to PARPi, with an optimal PFS of 39.8 months for mutations in the BRCA2 DNA binding domain and RAD51 binding domain (20). A *post-hoc* analysis of the PAOLA-1 study indicated that patients with BRCA1/2 mutations in different regions, particularly in the BRCA1/2 DNA binding domain, could benefit from maintenance therapy with olaparib plus bevacizumab (21). Several studies have shown that patients with mutations in the BRCA1 RING (Really Interesting New Gene) region are less sensitive to PARPi (22–24), suggesting that these mutations may contribute to resistance to both PARPi and platinum-based chemotherapy. In our study, frameshift mutations were observed in the BRCT domains of BRCA1, and one patient who received PARPi re-treatment had better efficacy with a PFS of 50.6 months compared to patients with other mutations. Conversely, if nonsense mutation occurs in the germline BRCA gene, the PFS was 3.3 months, 2.9 month, and 2.8 months respectively. This suggests that the presence of nonsense mutation in BRCA may be a factor contributing to PARP inhibitors resistance. However, the number of patients that could be analyzed in this study was limited, highlighting that larger-scale investigations are required to further explore this issue.

In the OReO/ENGOT Ov-38 study, the proportion of patients receiving rechallenge maintenance treatment with Olaparib who discontinued treatment was 24.7%. In contrast, no treatment termination was observed in our study and only 2 patients (3.9%) required interruptions in re-maintenance therapy due to TEAEs. This suggests a better safety profile of PARPi re-maintenance therapy in our cohort.

Overall, this study explores the use of PARPi in PSROC patients with prior PARP inhibitor exposure in the Chinese population. Although the study was limited in size and retrospective, it suggests that re-maintenance therapy with PARPi can still benefit patients who previously received PARPi treatment. Further research with larger sample sizes is needed to validate these findings. As the utilization of PARPi in first-line maintenance therapy for advanced OC and BRCA-related cancers increases, more patients will experience PARPi exposure and/or disease progression during PARPi therapy. Effective prediction of re-treatment efficacy and strategies to overcome acquired resistance will remain clinical priorities and require ongoing research. Limitations of this study include its single-center, retrospective nature, small sample size, and lack of randomized controls, all of which may reduce the validity of subgroup analyses. Longer follow-up is required to improve the comprehensiveness and reliability of survival data. In addition, further studies are needed to examine the mechanisms of PARPi resistance and their implications for subsequent therapeutic approaches.

## Conclusion

Re-treatment of PSROC patients with PARPi results in PFS benefit regardless of BRCA mutation status. Notably, the interval between PARPi treatments is a key factor affecting the efficacy of PARPi re-maintenance therapy. Structural domains of BRCA mutations with different sensitivity to PARPi may serve as a promising biomarker for a more effective treatment. Re-treatment with PARPi was tolerable.

## Data Availability

The datasets are stored in Research Data Deposit with an access number RDDA2024295288 (https://www.researchdata.org.cn/UserHome/). The datasets generated for this study are available on request to the corresponding authors.
